# Non-Contrast Fat-Saturated FLAIR MRI: Utility for Detecting Posterior and Seronegative-Associated Lesions in Optic Neuritis

**DOI:** 10.3390/diagnostics16183061

**Published:** 2026-09-21

**Authors:** Buhuan Zhang, Yuru Dong, Jinfeng Huang, Xuetao Mu

**Affiliations:** 1 Department of Radiology, 3rd Medical Center of Chinese PLA General Hospital, No. 69, Yongding Road, Haidian District, Beijing 100039, China; xjzhangbuhuan0991@163.com (B.Z.); dongyuru@sohu.com (Y.D.); 2 Department of Ophthalmology, 3rd Medical Center of Chinese PLA General Hospital, No. 69, Yongding Road, Haidian District, Beijing 100039, China; jinfeng1977@hotmail.com

**Keywords:** optic neuritis, magnetic resonance imaging, Aquaporin-4, Myelin Oligodendrocyte Glycoprotein, eye

## Abstract

**Background/Objectives:** To compare the lesion detection rates of fluid-attenuated inversion recovery with fat saturation (FLAIR-FS) versus T2-weighted short tau inversion recovery (T2WI-STIR) and contrast-enhanced T1-weighted imaging (CE-T1WI) in detecting lesions across antibody-defined subtypes (AQP4-ON, MOG-ON, seronegative ON) and anatomic segments of optic neuritis (ON). **Methods:** Forty-two patients (56 eyes) aged 5–57 years with acute/subacute optic neuritis underwent orbital MRI (T2WI-STIR, FLAIR-FS, and CE-T1WI). Two blinded neuroradiologists independently reviewed the images to assess lesion detectability and inter-observer reliability (kappa statistics). Generalized estimating equations (GEE) were used to account for within-patient correlation, with Bonferroni correction for pairwise comparisons. **Results:** FLAIR-FS demonstrated the highest detection rate (96.4%, 54/56 eyes), numerically higher than T2WI-STIR (80.4%, 45/56 eyes; corrected *p* = 0.051) and significantly higher than CE-T1WI (76.8%, 43/56 eyes; corrected *p* = 0.006). Inter-observer agreement was highest for FLAIR-FS (κ = 0.791), followed by CE-T1WI (κ = 0.729) and T2WI-STIR (κ = 0.681). In the posterior visual pathway, FLAIR-FS detected significantly more lesions than T2WI-STIR (corrected *p* = 0.024) and CE-T1WI (corrected *p* = 0.024). The Sequence × ON subtype interaction was not significant (*p* = 0.158); descriptively, FLAIR-FS showed the highest detection rate across all subtypes. **Conclusions:** FLAIR-FS demonstrated the highest lesion detection rate among the three sequences, particularly in the posterior visual pathway. FLAIR-FS should be regarded as a useful complementary non-contrast sequence, particularly when gadolinium cannot be administered.

## 1. Introduction

Optic neuritis (ON) is an inflammatory process involving the optic nerve [1], which is the most common cause of optic neuropathy in young adults. It can lead to acute or subacute vision loss, which seriously affects the quality of life of the patients [2,3]. Thus, early diagnosis and intervention are important. Currently, ON is internationally categorized into autoimmune and infectious/systemic ON (level 1), and this classification can be used to guide whether patients need long-term immunosuppressive therapy [4]. Autoimmune ON is an autoimmune reaction originating from optic nerve myelin and oligodendrocytes, and the pathological changes are mainly myelin loss [4]. It is classified according to “antibody type, anatomical localization, and disease course” into Aquaporin-4-ON (AQP4-ON), Myelin Oligodendrocyte Glycoprotein-ON (MOG-ON), and Single Isolated ON (SION), among others [4]. Seronegative ON is defined as ON that is negative for both AQP4-IgG and MOG-IgG, after exclusion of other identifiable causes. Two glial autoantibodies associated with demyelinating disease, AQP4 in neuromyelitis optica spectrum disorders (NMOSD) and MOG, are associated with unique clinical and radiologic features [1]. AQP4-ON may occur in the context of NMOSD and is characterized by severe visual loss, limited response to treatment, and poor recovery [5]. Numerous previous studies have shown that AQP4-ON is more often characterized by involvement of the optic chiasma, which is less frequently involved in other types of ON [6,7,8,9]. The explanation for the involvement of the optic chiasm by AQP4-ON is inconclusive, and it has been reported that it may be related to the high distribution of AQP4 in the optic chiasm [10]. In contrast, MOG-ON is characterized by frequent relapses that are steroid-responsive, and other CNS pathology is common [1]. The clinical and imaging manifestations of ON vary with different etiologic factors, which is of some significance for the diagnosis of the disease. Although ON can also represent the first manifestation of multiple sclerosis (MS), previous studies have focused more on MS [11,12,13], so seronegative ON, AQP4-ON, and MOG-ON were chosen for this study.

Magnetic resonance imaging (MRI) is increasingly used in the diagnosis of ON thanks to its high soft-tissue resolution and noninvasiveness, which clarify the site and scope of the lesion and provide supporting evidence for clinical diagnosis and differentiation [1,14,15], especially when the clinical symptoms are uncertain [16]. MRI is not only the gold standard for evaluating the presence and extension of signal and contrast-enhancement abnormalities of the optic nerve, but is also able to provide information useful to predict the visual outcome [11]. Features of ON on MRI include enhancement, abnormally high signal on T2WI and STIR [11], or swelling of the involved optic nerve [17]. Studies have shown that the median duration of persistent lesion enhancement in ON ranges from 63 to 113 days [17], while in acute demyelinating lesions of the brain, high-dose steroids can suppress gadolinium contrast enhancement within 1 day of treatment [18]. A previous small prospective study analyzing MRI findings in patients with the first episode of ON found that optic neuropathy was present in only 80.6% of cases [19]. MRI-negative ON is probably more common in routine clinical practice than these studies suggest. Another study showed that only 71.7% of routine MRI reports of patients with ON showed positivity (including T1WI with and without fat saturation, and T2WI with and without fat saturation) [16]. This discrepancy may lead some inexperienced clinicians to question the diagnosis of ON when MRI reports are normal, leading to unnecessary testing. The selection of an appropriate imaging sequence may improve the lesion detection rate of MRI for ON diagnosis to some extent.

Therefore, in this study, different MRI sequences of ON, including MOG-ON, AQP4-ON and seronegative-ON, were investigated to analyze the differences in the detection of ON by fluid-attenuated inversion recovery with fat saturation (FLAIR-FS), T2-weighted short tau inversion recovery (T2WI-STIR) and contrast-enhanced T1-weighted imaging (CE-T1WI), and to provide a reference for the subsequent selection of clinical diagnostic tools.

## 2. Methods

### 2.1. Patients

**Figure 1:**
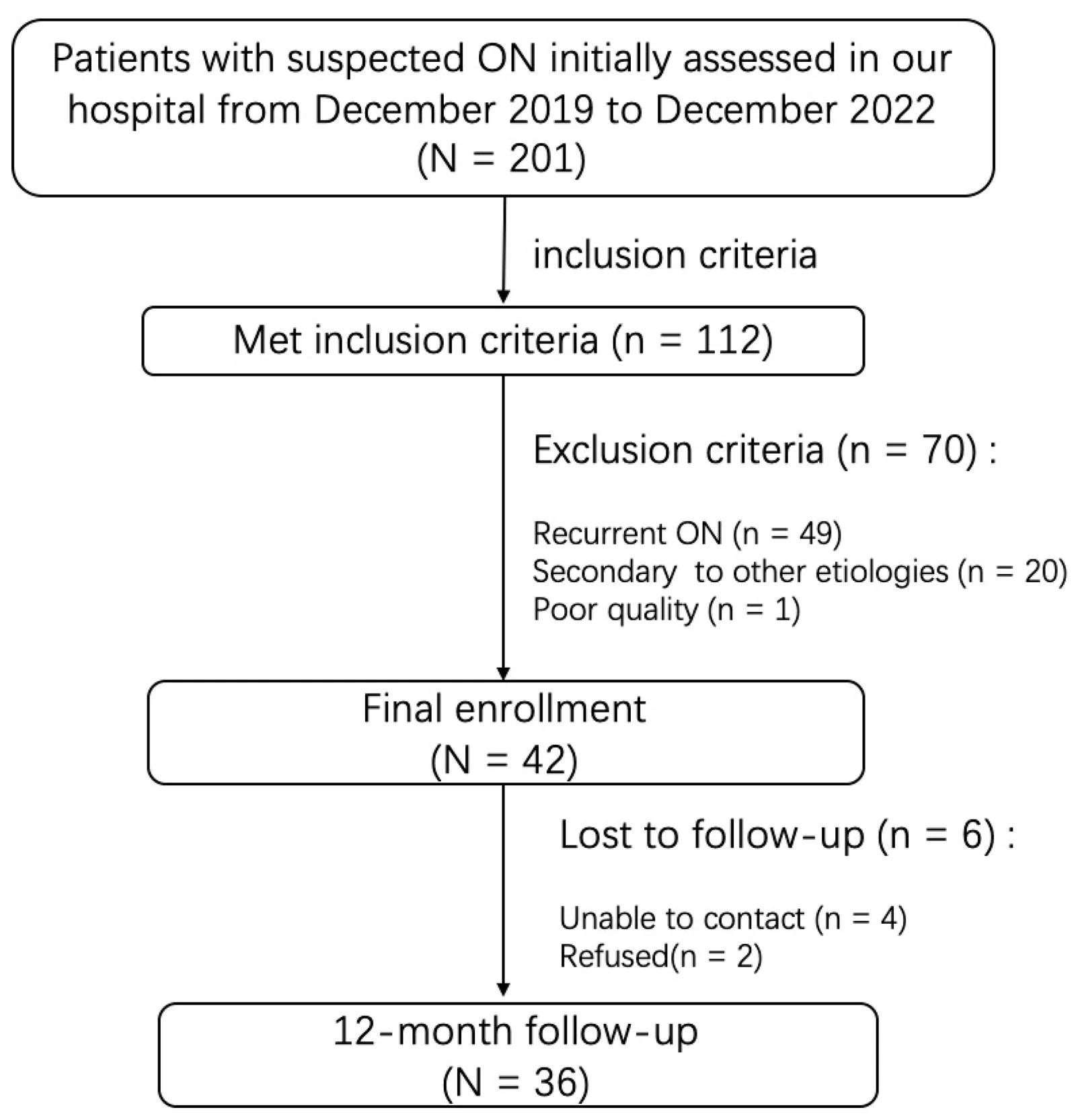
Flowchart of patient enrollment and 12-month follow-up.

Patients with ON in our hospital from December 2019 to December 2022 were consecutively enrolled. Inclusion criteria: (1) the diagnostic criteria of ON were in line with the criteria of the “Optic neuritis (2014)” [20] and “Diagnosis and classification of optic neuritis (2022)” [4]; (2) patients with clinical diagnosis of AQP4+/MOG+/seronegative ON; (3) the patient underwent orbital MRI examination, including T2WI-STIR/FLAIR-FS/CE-T1WI sequences, within 30 days of onset; (4) lesions confined to the optic nerve and/or optic tract, either alone or in combination. Exclusion criteria: (1) recurrent ON; (2) ON secondary to other etiologies, including but not limited to infectious optic neuropathy (e.g., syphilis, tuberculosis, viral infections, etc.), ischemic optic neuropathy, systemic inflammatory/autoimmune disorders, compressive optic neuropathy (e.g., tumors, thyroid-associated ophthalmopathy, etc.), nutritional/toxic optic neuropathy, inherited optic neuropathies (e.g., Leber’s hereditary optic neuropathy); (3) the quality of images is poor (e.g., motion artifacts). A flow diagram of patient enrollment and follow-up is shown in Figure 1. All patients underwent comprehensive baseline evaluations, including serum antibody testing for AQP4-IgG and MOG-IgG, as well as ophthalmological assessment (including BCVA and funduscopic examination) and orbital MRI. Other identifiable causes of optic neuritis were systematically excluded based on comprehensive clinical evaluation, laboratory work-up (including infectious serology, autoimmune antibodies, and genetic testing when clinically indicated), and imaging findings before patients were assigned to the seronegative ON group. In addition, information was collected on whether the patients had undergone steroid hormone therapy before MRI examination. At the 12-month follow-up, visual acuity was reassessed to determine visual recovery. This study was retrospectively approved by the ethics committee of the 3rd Medical Center of Chinese PLA General Hospital (KY2021-017) in accordance with the Helsinki Declaration of 1975, as revised in 2008.

### 2.2. Ophthalmological Examination

All patients underwent a comprehensive ophthalmological evaluation at presentation, including detailed medical history taking, best-corrected visual acuity (BCVA) measurement, and funduscopic examination. BCVA was measured using a standard Snellen chart and recorded in decimal notation. For eyes with vision too poor to be measured on the chart, visual acuity was categorized as counting fingers (CF), hand motion (HM), light perception (LP), or no light perception (NLP). Baseline BCVA was defined as the worst recorded visual acuity before discharge. Funduscopic findings were classified as normal (clear disc margins, pinkish disc coloration, and no hemorrhages or vascular abnormalities) or abnormal (presence of optic disc edema, hyperemia, blurred margins, peripapillary hemorrhage, venous tortuosity, or optic disc pallor). All examinations were performed by experienced ophthalmologists.

Visual recovery at 12 months was assessed using BCVA. Favorable recovery was defined as meeting either of the following criteria: (1) final BCVA was consistent with the pre-onset level; or (2) improvement from baseline BCVA as follows: ① if baseline BCVA was <0.1, final BCVA improved to >0.1; ② if baseline BCVA was ≥0.1, final BCVA improved by ≥0.3 logMAR (equivalent to ≥2 Snellen lines). Patients who met neither criterion were classified as having unfavorable recovery.

### 2.3. Laboratory Testing

Serum samples were collected from all patients at the time of acute presentation, prior to the initiation of corticosteroid therapy whenever possible. All samples were sent to Hangzhou Dian Medical Laboratory (Hangzhou, China), a nationally certified diagnostic facility, for antibody testing. AQP4-IgG and MOG-IgG were detected using a live cell-based assay (live CBA) according to the manufacturer’s instructions. Serum was used as the testing specimen, and a titer of ≥1:10 was defined as positive for both AQP4-IgG and MOG-IgG, based on the manufacturer’s recommended cut-off and published guidelines. Patients were classified into three subgroups based on antibody status: AQP4-ON (AQP4-IgG positive, MOG-IgGnegative), MOG-ON (MOG-IgG positive, AQP4-IgG negative), and seronegative ON (negative for both AQP4-IgG and MOG-IgG, after exclusion of other identifiable causes of optic neuritis). Antibody testing was performed at the time of the acute episode, and no repeat testing was performed for initially seronegative patients due to the retrospective nature of the study.

### 2.4. MRI Image Acquisition and Evaluation

#### 2.4.1. Images and Image Processing

All MRI examinations were performed on a 3-T scanner (Trio Tim, Siemens, München, Germany) using a 16-channel array head coil. The protocol included axial and coronal T2WI-STIR, FLAIR-FS, and CE-T1WI. Axial and coronal images were independently acquired for each sequence. CE-T1WI was acquired after intravenous injection of gadopentetate dimeglumine (Magnevist, Bayer Healthcare, Wayne, NJ, USA) at a dose of 0.2 mL/kg (0.1 mmol/kg), with scanning initiated 5 min after contrast administration. The detailed MRI acquisition parameters, including TR/TE/TI, turbo factor, voxel dimensions, image matrix, field of view, slice thickness, number of averages, fat-suppression technique, and acquisition time, are summarized in Supplementary Table S1. The protocol remained unchanged throughout the study period (2019–2022).

#### 2.4.2. Blinded Imaging Review

All MRIs were anonymized and patient identifiers were removed prior to review. MRIs were evaluated in a random order by two specially trained neuroradiologists (reader A with experience of 7 years and reader B with experience of 8 years). The readers were informed that the study cohort included patients with clinically suspected ON, and they were asked to determine whether an abnormal signal consistent with ON was present in each anatomical segment. They were blinded to the final clinical diagnosis, antibody status (AQP4-ON, MOG-ON, or seronegative ON), disease duration, treatment history, visual outcomes, and the results of the other sequences. Each sequence was evaluated in a separate reading session, with a minimum washout period of 4 weeks between sessions for the same patient to minimize recall bias. The order of sequence evaluation was generated using a computer-based random number generator (SPSS version 26.0, IBM Corp., Armonk, NY, USA), with a unique random sequence assigned to each patient. Signal abnormality in MRI was defined as increased signal intensity within the optic nerve compared with the normal white matter signal intensity. The visual pathway was divided into four anatomical segments for lesion localization: (1) orbital, (2) canalicular, (3) cisternal, and (4) chiasmal/optic tract. Each segment was evaluated independently for the presence of ON lesions. For the purpose of segment-based analysis, the four anatomical segments were further grouped into two composite categories: the anterior visual pathway (orbital and canalicular segments) and the posterior visual pathway (cisternal and chiasmal/optic tract segments). Each reader recorded for each sequence whether an ON lesion was present or not, using a binary decision (1 = detected, 0 = not detected). Final lesion detection for each sequence was determined by consensus between the two readers. In cases of disagreement, a third senior neuroradiologist adjudicated (reader C with 15 years of experience). A sequence was classified as “detected” if a lesion was identified by consensus review, and “not detected” if no lesion was identified.

### 2.5. Statistical Analysis

Descriptive statistics were used to summarize baseline patient demographics and lesion characteristics. Means and standard deviations (SD) were calculated for continuous variables; frequencies and percentages were calculated for categorical variables. Inter-observer agreement was assessed using Cohen’s kappa (κ) statistics based on binary lesion detection (lesion present vs. absent) for each sequence independently, with 95% confidence intervals and the following interpretation: 0.81–1.0 = “almost perfect”; 0.61–0.80 = “substantial”; 0.41–0.60 = “moderate”; 0.21–0.40 = “slight”; and 0.00–0.20 = “poor”. Given the absence of a sequence-independent gold standard, we use the term “lesion detection rate” rather than “sensitivity” or “true positive rate” throughout the manuscript. A total of 56 eyes (from 42 patients) were included in the statistical analysis. Given that chiasmal and optic tract involvement was included, these 56 eyes served as the anatomical units of observation. To account for within-patient correlation due to bilateral involvement, generalized estimating equations (GEE) with an exchangeable working correlation matrix were used, clustering by patient. Bonferroni correction was applied for the pairwise sequence comparisons. Results are reported as mean differences with 95% confidence intervals and corrected *p* values. For prognostic analysis, visual recovery at 12 months was assessed as a binary outcome (favorable vs. unfavorable). Given the limited sample size, multivariable logistic regression adjusting for ON subtype only was performed to assess whether posterior lesion location independently predicted visual recovery; results are reported as adjusted odds ratios (OR) with 95% confidence intervals. All statistical tests were performed at a two-tailed alpha level of 0.05. Data were analyzed using SPSS statistical software (version 26.0, IBM Corp., Armonk, NY, USA).

## 3. Results

### 3.1. Demographic and Clinical Characteristics

A total of 42 patients (56 optic nerves) were enrolled, ranging in age from 5 to 57 years, with more females than males (1.47:1). Twelve patients were diagnosed as AQP4-ON (28.6%), nine as MOG-ON (21.4%), and 21 were classified as seronegative ON (50.0%). Fourteen patients (33.3%) had bilateral lesions on MRI, and the remaining patients showed unilateral lesions. All patients had visual field defects, among which central and paracentral visual field defects were the most common. Other types of visual field defects included arc defects, high level defects, nasal defects, hemilateral visual field defects, and nonspecific defects. The mean time from symptom onset to MRI examination was 21.2 days (7–30 days). Ten patients (23.8%) received steroid hormone therapy before MRI examination. The detailed information on patients at baseline is shown in Table 1.

Twelve-month visual outcome data were available for 38 patients (48 eyes). In multivariable logistic regression adjusting for ON subtype, posterior lesion location remained significantly associated with unfavorable visual recovery at 12 months (adjusted OR = 0.216, 95% CI 0.047–0.991, *p* = 0.049). ON subtype was not a significant independent predictor in this model (*p* = 0.654).

### 3.2. Consistency Analysis of MRI Sequences

The consistency of lesion detection in ON patients for reviewer A and reviewer B on T2WI-STIR, FLAIR-FS, and CE-T1WI sequences was analyzed. Table 2 shows substantial inter-observer agreement for all three sequences, with FLAIR-FS (κ = 0.791) showing the highest consistency, followed by CE-T1WI (κ = 0.729) and T2WI-STIR (κ = 0.681). The underlying 2 × 2 reader-agreement tables for each sequence are provided in Supplementary Table S2.

### 3.3. MRI Sequence Detection of ON Lesions by Segment

The overall comparison among the three sequences, using a GEE model without segment stratification, showed a significant difference in detection rates (Wald χ^2^ = 11.51, df = 2, *p* = 0.003). In the GEE model including the Sequence × SegmentGroup interaction, the overall effect of Sequence remained significant (Wald χ^2^ = 14.48, df = 2, *p* = 0.001), and the interaction term was not significant (Wald χ^2^ = 3.85, df = 2, *p* = 0.146), indicating that the relative performance of the three sequences was consistent across anterior and posterior segments.

**Figure 2:**
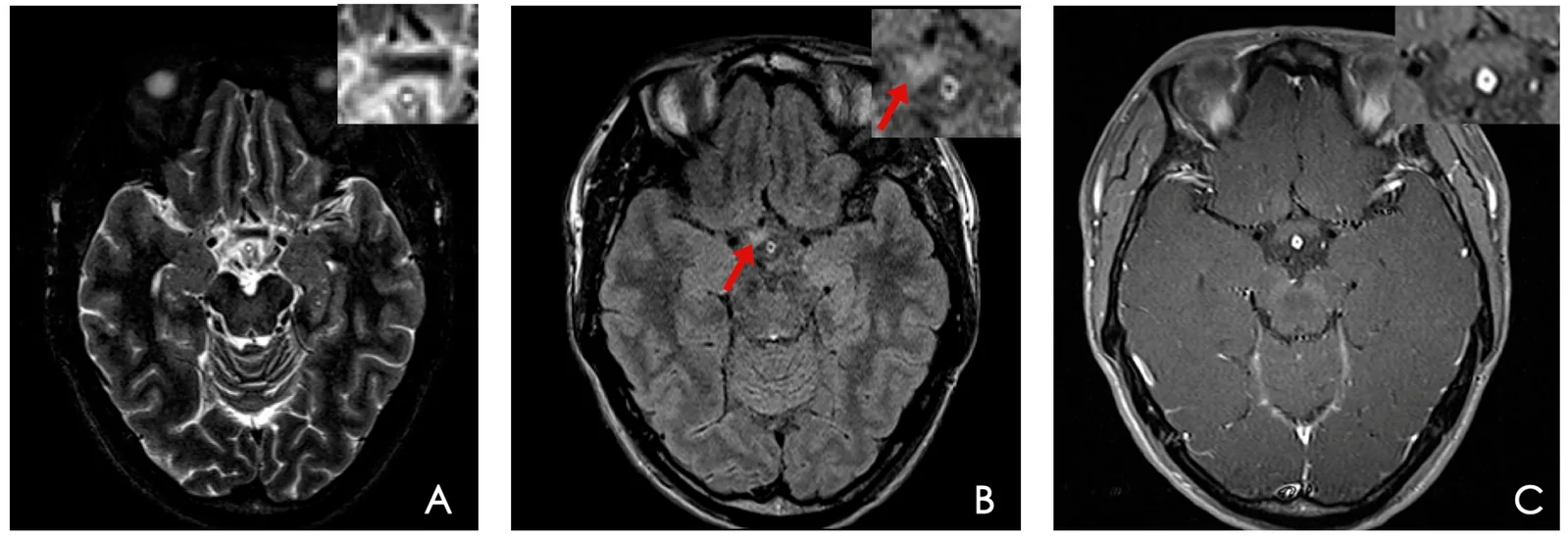
Seronegative ON with a lesion located at the optic chiasma. Axial T2WI-STIR (**A**) does not show a significant lesion; Axial FLAIR-FS (**B**) shows a localized high signal at the optic chiasma (red arrow); Axial CE-T1WI (**C**) shows no corresponding enhancement at the optic chiasma. All panels were displayed at identical magnification and windowing settings to facilitate direct comparison.

Pairwise comparisons with Bonferroni correction are summarized in Table 3. In the overall analysis, FLAIR-FS demonstrated a significantly higher detection rate than CE-T1WI (corrected *p* = 0.006) and showed a trend toward superiority over T2WI-STIR (corrected *p* = 0.051), whereas no significant difference was observed between CE-T1WI and T2WI-STIR (corrected *p* = 0.747). In the anterior segment, pairwise comparisons showed that FLAIR-FS was significantly superior to CE-T1WI (corrected *p* = 0.012), but not significantly different from T2WI-STIR (corrected *p* = 0.441). In the posterior segment, FLAIR-FS detected significantly more lesions than both T2WI-STIR (corrected *p* = 0.024) and CE-T1WI (corrected *p* = 0.024). No significant difference was observed between T2WI-STIR and CE-T1WI (corrected *p* = 1.000). Representative images from a patient with seronegative ON are shown in Figure 2.

### 3.4. Stratified Descriptive Analyses of MRI Sequences

Descriptive stratified analyses are shown in Supplementary Table S3. All three sequences showed higher detection rates in pediatric patients than in adults, and in those imaged within 14 days of symptom onset compared with those imaged later. FLAIR-FS and T2WI-STIR demonstrated higher detection rates in steroid-treated patients, whereas CE-T1WI showed higher detection in steroid-naïve patients. Notably, across all strata, FLAIR-FS consistently demonstrated the highest detection rate, suggesting that its advantage was stable across clinically relevant subgroups.

### 3.5. Differences in the Detection of Different ON Subtypes by MRI Sequences

To further explore whether the performance of the three sequences differed across ON subtypes, the GEE model including the Sequence × ON subtype interaction showed no significant interaction between sequence and ON subtype (Wald χ^2^ = 6.605, df = 4, *p* = 0.158), indicating that the relative performance of the three sequences was consistent across seronegative, AQP4-ON, and MOG-ON groups. Descriptively, FLAIR-FS showed the highest detection rate in all three subtypes, particularly in seronegative ON (27/27, 100%) (Table 4).

## 4. Discussion

In this study, the differences in the detection of acute or subacute ON by MRI sequences were analyzed. To standardize the assessment, axial and coronal images of each sequence were used for analysis. On the whole, the detection rate of ON by MRI sequences, including T2WI-STIR, FLAIR-FS and CE-T1WI, ranged from 76.8% to 96.4%, which is generally consistent with previous studies [21]. Notably, FLAIR-FS demonstrated the highest detection rate (96.4%, 95% CI 87.7–99.6%) among the three sequences across all ON subtypes. In the seronegative ON subgroup, FLAIR-FS detected more lesions than T2WI-STIR and CE-T1WI; however, given the limited sample size of this subgroup, this finding should be considered exploratory and requires confirmation in larger cohorts. Inter-observer agreement was substantial for all three sequences, with FLAIR-FS showing the highest consistency, followed by CE-T1WI and T2WI-STIR.

T2WI is one of the important routine MRI sequences showing the lesions of ON. Lesions of ON usually show abnormally high signals on T2WI. Its pathological mechanisms include inflammation, demyelination and gliosis of the optic nerve [22,23]. Since the fat around the optic nerve produces high signals and chemical shift artifacts, fat suppression is critical for T2WI. The cerebrospinal fluid wrapped by the optic nerve sheath appearing as high signal on T2WI sequences results in two issues: (1) The abnormal high signal of optic neuritis is not obvious due to the partial volume effect; (2) In the acute or subacute phase of ON, the optic nerve sheath is usually dilated, which reinforces the partial volume effect of cerebrospinal fluid. Therefore, the optimal T2WI sequence should inhibit both water and fat signaling. FLAIR, especially 3D-FLAIR with fat saturation, has a lesion detection rate of 100% in diagnosing acute ON [24,25]. In this study, the lesion detection rate of FLAIR-FS was 96.4% in diagnosing acute or subacute ON, which was numerically higher than that of T2WI-STIR (80.4%), although the difference did not reach the adjusted significance threshold after Bonferroni correction.

The optic nerve is a continuation of the brain and has a blood–optic nerve barrier similar to the blood–brain barrier. The enhancement of lesions is based on the contrast agent’s ability to pass through the damaged blood–optic nerve barrier, which is a characteristic MRI manifestation of acute or subacute ON. In our cohort, CE-T1WI demonstrated a lesion detection rate of 76.8%, which was comparable to T2WI-STIR and significantly lower than FLAIR-FS. Several factors may explain this observation. First, the timing of MRI acquisition relative to symptom onset is critical: gadolinium enhancement is time-sensitive and may wane as inflammation subsides. In our real-world clinical setting, MRI scheduling delays were not uncommon, which could have reduced the lesion detection rate of CE-T1WI in some patients. By contrast, fluid-attenuated inversion recovery with fat suppression, which does not rely on dynamic vascular permeability, may be less affected by the timing of imaging. Second, although treatment effects could theoretically influence enhancement patterns, the heterogeneity and incompleteness of treatment data in this retrospective cohort preclude a definitive analysis. Although we provided descriptive stratified analyses by timing of MRI and steroid pretreatment, the small sample size precluded formal adjusted modeling. We therefore acknowledge this as a limitation and emphasize that the lower detection rate of CE-T1WI should be interpreted with caution, and FLAIR-FS should be regarded as a useful complementary non-contrast sequence rather than a replacement for contrast-enhanced imaging. These findings should be validated in prospective studies with standardized imaging protocols and controlled treatment conditions.

Previous studies have reported that in the acute phase of ON, the length of optic neuropathy is an imaging biomarker for predicting retinal nerve axon loss and chronic visual impairment [13]. It has been reported that patients with ON involving the optic chiasma have a poor prognosis [6,7,8,9]. In line with these reports, our 12-month follow-up data demonstrated that patients with lesions located in the posterior visual pathway (cisternal, chiasmal, and optic tract) had poorer visual recovery than those with anterior involvement (orbital and canalicular). After adjusting for ON subtype, posterior lesion location remained associated with poorer visual recovery (adjusted OR = 0.216, 95% CI 0.047–0.991, *p* = 0.049), suggesting that the prognostic value of lesion location is not entirely explained by antibody status. However, due to the limited sample size, additional covariates including baseline visual acuity, age, and treatment could not be included in the model, and this finding should therefore be interpreted as an associated factor rather than an independent determinant. Larger prospective cohorts are needed to confirm this association.

We further evaluated the performance of each MRI sequence in detecting lesions at different anatomical locations. In the posterior visual pathway, FLAIR-FS detected significantly more lesions than T2WI-STIR and CE-T1WI, whereas no significant difference was observed between T2WI-STIR and CE-T1WI. This advantage may be attributed to the intrinsic tissue contrast provided by FLAIR-FS, which is not affected by perioptic cerebrospinal fluid signal, allowing better visualization of posterior optic nerve pathology. Furthermore, CE-T1WI was less effective than FLAIR-FS in visualizing lesions across all locations. Although the Sequence × SegmentGroup interaction was not statistically significant (*p* = 0.146), the numerical advantage of FLAIR-FS was most pronounced in the posterior visual pathway, where it detected 48.2% of lesions compared with 30.4% for T2WI-STIR and 33.9% for CE-T1WI. These findings suggest that FLAIR-FS may serve as a useful complementary non-contrast sequence, particularly for detecting lesions in the posterior visual pathway and in patients in whom gadolinium administration is contraindicated.

For the first episode of acute-phase ON, AQP4-ON is more likely to occur in females and usually shows unilateral lesions with significant enhancement, whereas MOG-ON is more likely to present with bilateral long-segment lesions. AQP4-ON is also more likely to involve the optic chiasma than MOG-ON and has a poorer prognosis [9]. In this study, both AQP4-ON and seronegative ON patients showed a numerical trend toward worse 12-month visual recovery compared with MOG-ON patients, although the difference did not reach statistical significance. The GEE model including the Sequence × ON subtype interaction showed no significant interaction, indicating that the relative performance of the three sequences was consistent across seronegative, AQP4-ON, and MOG-ON groups. Descriptively, FLAIR-FS showed the highest detection rate in all three subtypes, with this numerical advantage being most evident in the seronegative ON subgroup. However, given the limited sample size of this subgroup, these findings should be considered exploratory and require confirmation in larger cohorts. These findings highlight FLAIR-FS as a potentially useful imaging tool, particularly in seronegative ON, a subgroup with limited biomarker support. The higher detection rate of FLAIR-FS in this challenging population may aid in early lesion identification, although prospective validation is needed before clinical decision-making can be guided by these findings.

In addition to its retrospective single-center design, this study has some limitations. First, the sample size was relatively small, particularly after stratification by ON subtype, age group, anatomical segment, etc., which limited our ability to perform detailed subgroup analyses and may have contributed to the lack of statistical significance in certain comparisons. The cohort included both pediatric and adult patients; pediatric ON differs from adult ON in etiological distribution, bilateral presentation, MOG association, and clinical course. Although we provided a descriptive analysis by age group, the small number of pediatric patients precluded formal statistical comparison, and the potential influence of combining pediatric and adult ON should therefore be acknowledged. Second, contrast-enhanced sequences are not fully comparable with FLAIR or STIR, as gadolinium enhancement is transient and may wane over time, whereas T2-weighted abnormalities tend to persist longer. To mitigate this confounding effect, we excluded patients with chronic ON and restricted the cohort to those imaged within 30 days of symptom onset. However, the MRI acquisition time ranged from 7 to 30 days after symptom onset; this variability was not accounted for in our analyses, and the evolving nature of lesions over this period may have influenced sequence-specific detectability. Third, patients had received various treatments prior to MRI, which may have affected contrast enhancement patterns; however, data on prior treatment regimens were not completely available in this retrospective cohort. While the primary objective of this study was to compare the lesion detection rates of each sequence rather than to evaluate treatment effects, we acknowledge that this limitation should be considered when interpreting the findings. Fourth, repeat testing was not performed for initially seronegative patients in this retrospective cohort, which may have led to misclassification of some patients with delayed antibody seroconversion. Finally, this study evaluated only the comparative lesion detection rates among sequences in a cohort with a clinical diagnosis of ON. Because no disease-negative control group was included, we could not assess specificity, predictive values, or overall diagnostic accuracy. Future studies with appropriate controls are needed to establish the full diagnostic performance of FLAIR-FS in the broader work-up of optic neuropathies.

## 5. Conclusions

In this selected cohort of patients with acute/subacute ON, FLAIR-FS demonstrated the highest lesion detection rate among the three sequences (FLAIR-FS, T2WI-STIR and CE-T1WI), with its advantage being particularly evident in the posterior visual pathway. These findings suggest that FLAIR-FS may serve as a useful complementary non-contrast sequence, particularly when gadolinium cannot be administered. However, because this study did not include controls and did not assess specificity, FLAIR-FS cannot be considered a replacement for CE-T1WI, which provides additional information regarding the integrity of the blood–optic nerve barrier. Prospective diagnostic-accuracy studies including appropriate controls are needed to establish the full diagnostic utility of FLAIR-FS in the broader work-up of optic neuropathies.

## Figures and Tables

**Table 1:** Clinical characteristics of patients with optic neuritis.

Item		Value
Patients		*N* = 42
Age (years)		35.2 ± 15.3 (5–57)
Age group, *n* (%)	Children (<18 yrs), *n* (%)	9 (21.4)
	Adult (≥18 yrs), *n* (%)	33 (78.6)
Gender (cases)		
	Male, *n* (%)	17 (40.5)
	Female, *n* (%)	25 (59.5)
	Female: Male	1.47:1
Subgroups (cases)		
	seronegative-ON, *n* (%)	21 (50)
	AQP4-ON, *n* (%)	12 (28.6)
	MOG-ON, *n* (%)	9 (21.4)
Location (cases)		
	left, *n* (%)	10 (23.8)
	right, *n* (%)	18 (42.9)
	bilateral, *n* (%)	14 (33.3)
The mean time from symptom onset to MR examination (days)		21.2 ± 7.3 (7–30)
	≤14 days, *n* (%)	11 (26.2)
	>14 days, *n* (%)	31 (73.8)
Symptom onset (cases)		
	Loss of vision, *n* (%)	39/42 (92.9)
	Eye pain, *n* (%)	21/42 (50.0)
Steroid hormone therapy before MR examination (cases)		
	yes, *n* (%)	10 (23.8)
	no, *n* (%)	32 (76.2)
Eyes		*N* = 56
Funduscopic findings		
	Normal, *n* (%)	31 (55.4)
	Optic disc abnormality, *n* (%)	25 (44.6)
Baseline BCVA		
	>0.5, *n* (%)	6 (10.7)
	0.1~0.5, *n* (%)	10 (17.9)
	<0.1 ^#^, *n* (%)	40 (71.4)
12-Month visual outcome *		*N* = 48
	Favorable recovery, *n* (%)	32 (66.7)
	Unfavorable recovery, *n* (%)	16 (33.3)

^#^ BCVA < 0.1 included counting fingers (CF), hand motion (HM), light perception (LP), and no light perception (NLP). * The 12-month visual outcome was assessed in 48 eyes (36 patients), and percentages for favorable/unfavorable recovery are based on the 48 eyes with available follow-up data. Abbreviations: ON, optic neuritis; AQP4, Aquaporin-4; MOG, Myelin Oligodendrocyte Glycoprotein; BCVA, best-corrected visual acuity.

**Table 2:** Consistency analysis of MR sequences by different readers.

Readers	T2WI-STIR	FLAIR-FS	CE-T1WI
reader A	38	53	39
reader B	45	54	43
κ (95% CI *)	0.681 (0.471–0.891)	0.791 (0.393–1.000)	0.729 (0.529–0.929)
*p*	<0.001	<0.001	<0.001

* 95% CI calculated as κ ± 1.96 × asymptotic standard error.

**Table 3:** Detection of lesions in different segments by different MR sequences.

Segment	Sequence	Detection Rate, % (*n*/*N*) [95% CI] ^a^	Pairwise Comparison	Mean Difference (95% CI) ^b^	*p* Value (Bonferroni-Corrected) ^c^
Overall *	FLAIR-FS	96.4 (54/56) [87.7–99.6]	FLAIR-FS vs. T2WI-STIR	0.12 (0.02, 0.22)	0.051
	T2WI-STIR	80.4 (45/56) [68.2–89.0]	FLAIR-FS vs. CE-T1WI	0.19 (0.07, 0.31)	**0.006**
	CE-T1WI	76.8 (43/56) [64.2–86.1]	T2WI-STIR vs. CE-T1WI	0.07 (−0.05, 0.19)	0.747
Anterior	FLAIR-FS	82.1 (46/56) [70.0–90.2]	FLAIR-FS vs. T2WI-STIR	0.05 (−0.02, 0.11)	0.441
	T2WI-STIR	76.8 (43/56) [64.2–86.1]	FLAIR-FS vs. CE-T1WI	0.17 (0.05, 0.28)	**0.012**
	CE-T1WI	66.1 (37/56) [52.9–77.3]	T2WI-STIR vs. CE-T1WI	0.12 (0.00, 0.24)	0.144
Posterior	FLAIR-FS	48.2 (27/56) [35.5–61.2]	FLAIR-FS vs. T2WI-STIR	0.14 (0.04, 0.25)	**0.024**
	T2WI-STIR	30.4 (17/56) [19.7–43.6]	FLAIR-FS vs. CE-T1WI	0.14 (0.04, 0.25)	**0.024**
	CE-T1WI	33.9 (19/56) [22.5–47.5]	T2WI-STIR vs. CE-T1WI	0.00 (−0.11, 0.11)	1.000

* Overall comparisons were derived from a separate GEE model without segment stratification. ^a^ Data are presented as raw detection rates with 95% Wilson confidence intervals. ^b^ Mean differences and 95% confidence intervals are derived from the GEE model with robust standard errors, comparing estimated marginal means between sequences within each segment group. ^c^ Bonferroni correction was applied for pairwise comparisons, and bold values indicate statistical significance after correction.

**Table 4:** Detection of different ON subgroups with different MR sequences.

Parameters	*n* (Eyes)	T2WI-STIR	FLAIR-FS	CE-T1WI
seronegative-ON	27	21 (77.8%)	27 (100%)	19 (70.4%)
AQP4-ON	16	15 (93.8%)	15 (93.8%)	14 (87.5%)
MOG-ON	13	9 (69.2%)	12 (92.3%)	10 (76.9%)

Abbreviations: ON, optic neuritis; AQP4, aquaporin-4; MOG, myelin oligodendrocyte glycoprotein.

## Data Availability

The data presented in this study are available on request from the corresponding author due to privacy and ethical restrictions.

## Supplementary Materials

The following supporting information can be downloaded at https://www.mdpi.com/article/10.3390/diagnostics16183061/s1, Table S1. MRI Acquisition Parameters. Table S2. 2 × 2 reader agreement tables for each MRI sequence. (a) T2WI-STIR. (b) FLAIR-FS. (c) CE-T1WI. Table S3. Descriptive stratified lesion detection rates of three MRI Sequences.
