# Supplementary material for: Non-Contrast Fat-Saturated FLAIR MRI: Utility for Detecting Posterior and Seronegative-Associated Lesions in Optic Neuritis
Source: Diagnostics (Basel). 2026 Sep 21;16(18):3061. doi: 10.3390/diagnostics16183061 (PMC13605796; doi:10.3390/diagnostics16183061)
Supplement: Supplementary file 1 [file diagnostics-16-03061-s001.zip › diagnostics-4546390-supplementary.pdf]

Supplementary Tables

Supplementary Table S1. MRI Acquisition Parameters

Parameter	T2WI-STIR	FLAIR-FS	CE-T1WI
Acquisition type	2D	2D	2D
Acquisition plane	axial and coronal	axial and coronal	axial and coronal
TR(ms)	5860ms	8000ms	530 ms
TE(ms)	107ms	85ms	8.2ms
TI(ms)	220ms	2720ms	-
Turbo factor	18	16	-
voxel dimensions(mm)	0.74x0.59x2	0.85x0.59x2	0.9x0.7x2
Image matrix	256x320	256x320	212 × 256
Field of view (mm)	190 × 190	190 × 190	190 × 190
Slice thickness (mm)	2.0	2.0	2.0
number of averages	2	1	1
exact fat-suppression technique	STIR	FS	FS
acquisition time(min:sec)	3:03	2:58	3:21

Abbreviations: TR, Repetition Time; TE, Echo Time ;TI, Inversion Time ; STIR, short tau inversion recovery; FS, Fat Saturation.

Supplementary Table S2. 2×2 reader agreement tables for each MRI sequence

S2a. T2WI-STIR

	Reader B: detected	Reader B: not detected	Total
Reader A: detected	38	0	38
Reader A: not detected	7	11	18
Total	45	11	56

κ = 0.681 (95% CI: 0.471–0.891), P < 0.001

S2b. FLAIR-FS

	Reader B: detected	Reader B: not detected	Total

Reader A: detected	53	0	53
Reader A: not detected	1	2	3
Total	54	2	56

κ = 0.791 (95% CI: 0.393–1.000), P < 0.001

S2c. CE-T1WI

	Reader B: detected	Reader B: not detected	Total
Reader A: detected	38	1	39
Reader A: not detected	5	12	17
Total	43	13	56

κ = 0.729 (95% CI: 0.529–0.929), P < 0.001

Supplementary Table S3. Descriptive stratified lesion detection rates of three MRI Sequences

**Stratum**	**Subgroup**	**N (eyes)**	**FLAIR-FS,n/N(%)**	**CE-T1WI,n/N(%)**	**T2WI-STIR,n/N(%)**
Age	Children (<18 yrs)	14	14/14 (100.0)	14/14 (100.0)	12/14 (85.7)
	Adult (≥18 yrs)	42	40/42 (95.2)	29/42 (69.0)	33/42 (78.6)
Time from symptom onset to MR examination	Early (≤14 days)	17	17/17 (100.0)	15/17 (88.2)	15/17 (88.2)
	Late (>14 days)	39	37/39 (94.9)	28/39 (71.8)	30/39 (76.9)
Steroid hormone therapy before MR examination	No	42	40/42 (95.2)	34/42 (81.0)	31/42 (73.8)
	Yes	14	14/14 (100.0)	9/14 (64.3)	14/14 (100.0)

Stratified analyses are descriptive only; no statistical comparisons were performed across strata due to the limited sample size after stratification.
